# Eating and Drinking

**Published:** 1876-12

**Authors:** 


### EATING AND DRINKING.

Half knowledge is the source of a large number of the errors and discomforts in practical life. We must have both the knowledge and the reason of a thing in order to derive the highest advantage from our intelligence. The consciousness of a mere fact is comparatively valueless, besides it is hard to remember. Knowing the reason of a thing aids very much in impressing it on the mind. Our domestic directions and instructions should be oftener imparted in this manner than they are.

We wish the habitual readers of our Journal to grow up intelligent on health subjects. If we state a fact, or lay down a rule without a reason for it, some other person may give advice in direct variance, and the reader be thus left in a state of betweenity, or he may give a reason, adequate in his own view, and thus mislead.

We have advised persons that it is not well to drink cold water, or any other fluid, largely at meals ; if we must drink something, it should be warm rather than cold, especially if one is an invalid. A sip or two of cold water, now and then during a meal, is admissible to persons in good health. We have lately seen a statement from *Orr's Chemistry of Food and Diet*, that drinking during meals is *not* an obnoxious habit. If the reader will look with us into a man's stomach during a meal, he can perhaps judge as well as we can as to the facts in the case. This ocular view of the stomach has been afforded to medical men at different times, and the facts are not to be disputed. A gun loaded with buck shot was accidentally discharged into the stomach, the parts healed, leaving a cavity which gave facilities for observation.

The temperature of the stomach is about ninety-six degrees. A glass of cold water is about forty degrees more or less. When it is introduced into the stomach through the throat, it must diminish its temperature. But digestion cannot go on unless there is a heat of upwards of ninety degrees in the stomach ; hence when a man who is eating or has just eaten his dinner, drinks a glass of cold water, the process of digestion is seen to be arrested as suddenly as water ceases to boil when cold water is added, and the power of digestion remains arrested, until the water has been long enough in the stomach to acquire an additional heat of some fifty degrees. This heat is abstracted from the body. Thus it is that persons of weak health on going to the table of a summer's day, abundantly warm, leave it in a chilly condition, from having partaken largely of cold water. Such is the result sometimes from an ordinary meal, in persons of poor health, without drinking anything cold. If the meal be a hearty one, and the person has but little vitality, and goes out in the cold soon after, he will most probably lose his life. This was the manner of the death of the Duke of Wellington. He ate largely of venison, of a cold, damp, drizzly November day; the stomach could not get heat enough out of the body to carry on digestion. The system was loaded down, chilled, and oppressed, inducing stupor and eventuating in death next day perhaps. We know a gentleman of regular habits, who watched over himself with extraordinary care, approaching his eightieth year, J. W. S., the associate and personal friend of HENRY CLAY, a half a century before. He ate a hearty breakfast and walked to his office, two miles distant, the thermometer near zero, with a cutting wind from the river, and nothing to break its force; this double draft upon his vitality, or rather treble one, the walk in a wind, the coldness of the atmosphere, and the full stomach, at his great age—the assets—the supply of warmth, was overdrawn; in other words, he was thoroughly chilled, fell into a kind of stupor and died in two or three days. Therefore, exposure to severe cold immediately after eating, is always dangerous. The slightest chilliness after a meal, should drive any one to the fire, there to remain until it has passed entirely away. With these indisputable facts before him, the reader must see that drinking freely of cold water at meals, immediately arrests digestion, arrests a natural process of the bodily functions; and to arrest nature in such a work cannot be beneficial, and to persons not in full health has been fatal, and may be so again.

To invalids we say, if you drink anything at all at your meals, let it be hot, and not much of that even. An ordinary teacup full is enough, for it is seen that when any fluid is drank at or soon after meals, even water, the watery particles must be removed from the stomach before the process of digestion is resumed.

